# DNA direct reversal repair and alkylating agent drug resistance

**DOI:** 10.20517/cdr.2020.113

**Published:** 2021-06-19

**Authors:** Roberto Gutierrez, Timothy R. O’Connor

**Affiliations:** ^1^Department of Cancer Biology, Beckman Research Institute, City of Hope, Duarte, CA 91010, USA.; ^2^Irell & Manella Graduate School of Biological Sciences, City of Hope, Duarte, CA 91010, USA.; ^3^Department of Cancer Biology, Beckman Research Institute, City of Hope, Duarte, CA 91010, USA.

**Keywords:** Direct reversal repair, O6-methylguanine-DNA methyltransferase, AlkB homologs, resistance to alkylating agents

## Abstract

DNA direct reversal repair (DRR) is unique in that no DNA synthesis is required to correct the error and therefore repair via such mechanisms are error-free. In humans, DRR is carried out by two different pathways: the O6-methylguanine-DNA methyltransferase (MGMT) and the alkylated DNA repair protein B (AlkB) homologs. The use of alkylating agents is the standard of care for many cancers. However, the use of those drugs is usually halted when resistance develops. This review will examine repair of alkylating agent damage mediated by DRR, resistance mechanisms and potential ways to overcome such resistance.

## Introduction

DNA alkylating agents are still used for the treatment of many cancers. However, continued treatment with alkylating agents, even in drug cocktails, generally results in drug resistance. Increasing the utility of those drugs requires the understanding of the sources that oppose the therapeutic effects of alkylating agents. One of the principal mechanisms that contributes to alkylating agent resistance is DNA repair.

DNA alkylation damage occurs at all bases. The level of DNA damage at the individual bases does not correlate with the biological impact of the damage. Major damage sites at N7 of guanine does not generally cause mutation or cell death associated with therapeutic impact. More minor sites of alkylation damage, including O6 of guanine, N1 of adenine, or N3 of cytosine are more closely aligned with therapeutic responses of alkylating agents^[1,2]^.

**Figure 1:**
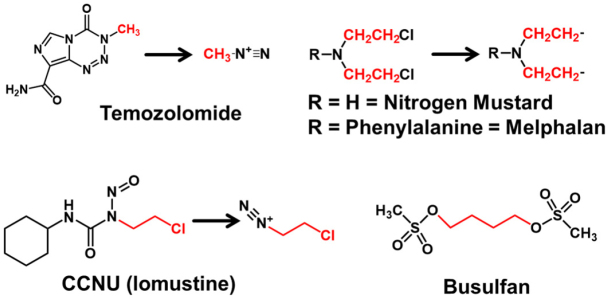
Selected chemotherapeutic alkylating agents. DNA damage is monofunctional (temozolomide, CCNU) or bifunctional (nitrogen mustard, melphalan, busulfan). Alkylating agents use S_N_1 (temozolomide, CCNU) or S_N_2 mechanisms (nitrogen mustard, melphalan, busulfan). The red portions of the structure are the moieties that alkylate DNA.

The number of alkylating agents used in chemotherapy is too long to cite in this brief review. To provide some examples, there are monofunctional methylating agents (temozolomide or TMZ), larger monofunctional alkylating agents (cyclohexyl chloroethyl nitrosourea, CCNU, or lomustine) [Figure 1]. There are bifunctional agents that interact and can cause inter- or intrastrand crosslinks (nitrogen mustards or busulfan) [Figure 1]. S_N_1 alkylating agents (e.g., temozolomide, lomustine in Figure 1) form reactive intermediates that then react with DNA according to kinetics dependent only on the concentration of alkylating agent, whereas S_N_2 alkylating agents (e.g., nitrogen mustard, busulfan in Figure 1) react directly with DNA and manifest kinetics that depend on the concentration of both the alkylating agent and the target. DNA that is damaged by these agents cause is restored to normal by mechanisms of DNA repair.

**Figure 2:**
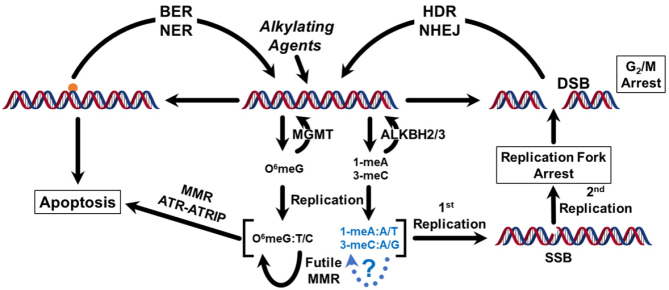
General DNA repair pathways implicated in the elimination of DNA damage from alkylating agents. The blue sections and dotted lines have not yet been described in the literature. The possible base pairs for O^6^meG, 1-meA, and 3-meC are indicated. If cells complete the first round of replication, following a second round of replication, a mutation is fixed in 50% of the cells. If replication is arrested and a second round of replication occurs, a DSB can form. Modeled after Reference^[3]^. BER: Base excision repair; NER: nucleotide excision repair; MMR: mismatch repair; DSB: double-strand break; HDR: homology-directed repair; NHEJ: non-homologous end-joining; SSB: single strand break; ATR: ataxia telangiectasia and Rad3 related; ATRIP: ATR interacting protein; O^6^meG: O^6^methylguanine; 1-meA: 1-methyladenine; 3-meC: 3-methylcytosine.

DNA repair is often collectively referenced, but DNA repair consists of numerous pathways that can restore genomic integrity and depend on the type of damage inflicted by an agent. Thus, detailing the ways that tumors develop resistance ultimately will require understanding how the ensemble of these pathways function together to protect cells [Figure 2]. Modification of DNA bases can lead to the activation of these pathways, depending on how the initial insult is addressed. Base or nucleotide excision repair can directly eliminate the damage. If damage levels are too high, cells can undergo apoptosis. Another possibility is the formation of double-strand breaks that can cause cell death. The simplest type of repair that will be described in this review is direct reversal repair (DRR), which does not require any DNA synthesis and is therefore error-free. DRR is conducted by O6-methylguanine-DNA methyltransferase (MGMT) and the alkylated DNA repair protein B (AlkB) homologs ALKBH2 and ALKBH3. Failure to repair prior to replication can result in an alternative DNA base that can lead to mutations. If the alternative base pairs are maintained there is a possibility that mismatch repair (MMR) will play a role in cell death. However, if replication is not continued through the mispairs, arrest of the replication fork can lead to a double-strand break that would be lethal if left unrepaired. To limit the scope of this review, we will concentrate on resistance associated with one type of DNA repair, DRR.

## O6-methylguanine-DNA methyltransferase pathway

**Figure 3:**
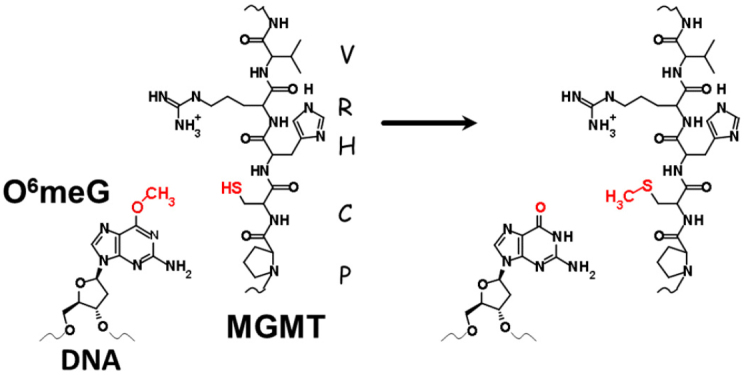
Transfer of methyl group from O^6^meG to the cysteine residue of MGMT. Conserved amino acids around the active site are shown. MGMT: O6-Methylguanine-DNA Methyltransferase; O^6^meG: O^6^methylguanine.

The DRR MGMT pathway is found in all cells. The reaction mechanism is a direct transfer of the alkylated base to the cysteine-145 receptor in MGMT in the conserved 5-amino acid active site PCHRV [Figure 3]^[4,5]^. That transfer is accompanied by ubiquitination of MGMT and subsequent degradation of the protein in the 26S proteasome^[6]^. In this repair, a single MGMT protein repairs a single O^6^-methylguanine (O^6^-meG), which represents a substantial energy requirement to remove a single damage from a base^[7]^.

**Figure 4:**
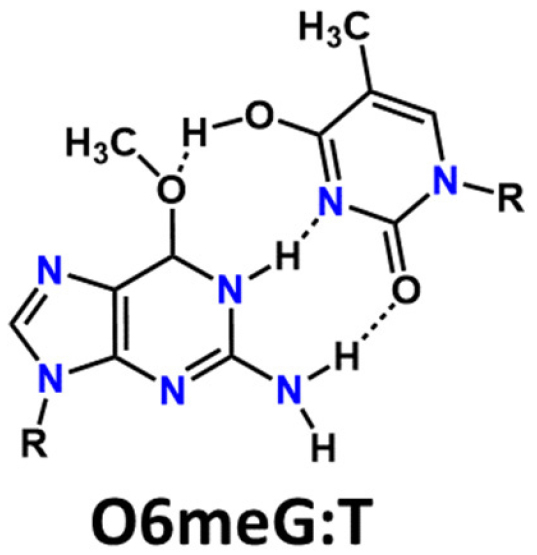
O^6^methylguanine:thymine base pairing that leads to G→A mutation. The pairing is based on data from the crystal structure of the pair in oligodeoxyribonucleotides^[8]^. Note that the thymine tautomer formed is not the standard tautomer drawn for thymine in Watson-Crick base pairs.

Persistence of O^6^meG during replication, can lead to the incorporation of thymine opposite O^6^meG. If no repair occurs and replication is completed the O^6^meG:T base pairing can form [Figure 4]. A subsequent round of replication leads to a G→A mutation.

### Persistent O^6^methylguanine lesions and cell death

When replication is finished, the base pair depicted in Figure 4 can be recognized by an active MMR pathway. If the MMR pathway removes the thymine, another thymine could be reinserted in a futile repair process. Repeated futile repair cycles eventually result in the cell undergoing apoptosis [Figure 2].

Although MGMT inhibitors can deplete MGMT activity, another way to reduce the MGMT levels is to take advantage of the variations in the levels of MGMT found in different cells. In many tumors, MGMT deficiency is linked to epigenetic methylation of the MGMT promoter region to produce 5-methylcytosines that silence expression^[9-11]^. MGMT promoter silencing is frequently found in tumors^[12,13]^. Tumors with low MGMT levels usually produce positive patient outcomes.

Alternatives to silencing MGMT expression in tumors have used drugs, such as O6-benzylguanine, which deplete MGMT protein levels by inactivating the protein. These MGMT inhibitors inactivate the protein and are still undergoing clinical evaluation. One potential consideration for the poor outcomes using MGMT inactivation is that drugs that deplete MGMT could also require other DNA repair systems, as noted for the futile repair cycles.

### Resistance to alkylating agent damage by MGMT

The levels of MGMT in tissues and in tumors vary greatly^[14,15]^. Tumors with low or MGMT-deficiency respond better to dacarbazine or temozolomide therapy than do tumors with MGMT levels similar to those found in normal cells^[16-21]^. Therefore, tumors with MGMT levels approximating those of normal cells are more resistant to alkylating agent treatment. Conversely, tumors with low MGMT levels respond more favorably to treatment as compared to tumors with normal MGMT levels^[17,22-24]^. Thus, improved outcomes for patients with lower MGMT levels indicate that normal MGMT levels provide resistance to dacarbazine or temozolomide treatment.

### Resistance to alkylating agent damage by MGMT in concert with mismatch repair

MGMT protects against alkylating agent damage, and the discovery that tumors and cell lines can spontaneously have the *MGMT* promoter silenced, certainly suggested that there could be cumulative effects that would result in further sensitization of tumors to alkylating agent treatments. The MMR system is another major DNA repair pathway that is often defective in tumors^[25]^. Hereditary nonpolyposis colon cancer has defective MMR genes that result in genomic instability^[26-28]^, but other tumor types can also evolve MMR deficiencies by mutations, although also by epigenetic silencing, particularly of *MLH1*^[29-34]^. The sensitivity of MMR-proficient cells in the absence of MGMT is due to the futile MMR cycles^[35,36]^ that are induced by O^6^meG:T mispairing [Figures 2 and 3]. The role of MMR was demonstrated using Msh2-deficient mouse embryonic stem cells and O6-benzylguanine that inactivates MGMT^[37]^. Further mouse model work demonstrated the resistance to alkylating agents that arises with the simultaneous inactivation of both the *Mgmt* and *Mlh1* and the increase in mutations upon treatment as compared to either of the genes inactivated independently^[38,39]^.

## AlkB homolog pathway

**Figure 5:**
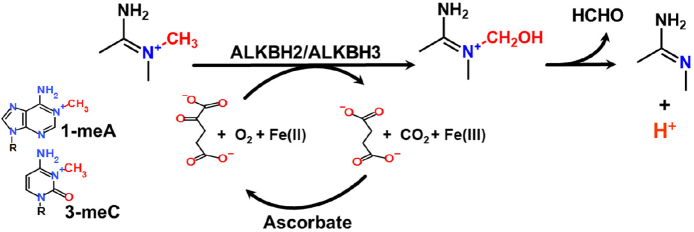
Repair of 1-methyladenine and 3-methylcyotsine. The reaction converts α-ketoglutarate to succinate with CO_2_ release and requires oxygen and Fe(II). There is also a release of formaldehyde to regenerate the normal base. 1-meA: 1-methyladenine; 3-meC: 3-methylcytosine.

The AlkB homolog (ALKBH) pathway in this review refers to both ALKBH2 and ALKBH3 (ALKBH), which reversibly remove numerous modified bases but principally 1-methyladenine (1-meA) and 3-methylcytosine (3-meC)^[40-42]^. There are 9 proteins among the AlkB homologs, but the functions for most are diverse and include removal of RNA modifications^[43]^. Numerous protein groups use similar reaction mechanisms, which include proline hydroxylation and ten-eleven translocation^[44-47]^. The ALKBH reaction mechanism uses an oxidative demethylation with a Fe(II) that is coordinated to amino acids in the proteins, along with α-ketoglutarate. The removal of the methyl group involves the conversion of α-ketoglutarate to succinate with the release of carbon dioxide and formaldehyde [Figure 5].

**Figure 6:**
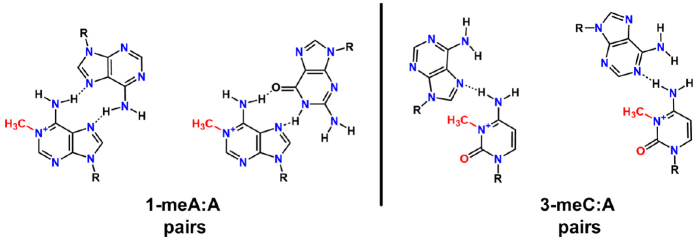
1-methyladenine:adenine and 3-methylcytosine:adenine base pairings, which lead to A→T and C→T mutations, respectively. These possible base pairing schemes are derived from the observation of mutations in a mouse model system with Alkbh2 or Alkbh3 deleted^[48]^. The 3-methylcytosine (3-meC) modified base would most probably use a translesion DNA polymerase for insertion opposite 3-meC due to the single-base pairing available with the modification at the N3 position. Note that the left 1-meA:A pair would be in a parallel helix, whereas the right structure would be in an antiparallel helix.

Persistence of 1-meA and 3-meC can lead to mutations that are reported to be A→T transversions or C→T transitions^[48]^, which involve 1-meA pairing with T or possibly bypass of the 3-meC, because only a single base pair can form with the 1-meC modification [Figure 6]. The bypass of C by translesion DNA polymerases inserting A opposite to produce a C→T transition mutation is suggested by an *in vitro* steady state kinetics study that showed a substrate preference for insertion of dATP, based on the k_cat_/K_M_ ratio^[49]^.

### Persistent 1-methyladenine and 3-methylcytosine lesions and cell death

Currently, the consequences of the completion of replication with the base pair structures in Figure 6 is unknown. On the basis of the link between MGMT and MMR, it is possible that mispairing occurs that leads to cell death [Figure 2], but that is not reported. Moreover, understanding the links between the MGMT and ALKBH pathways is important to the understanding of how tumor cells protect themselves against alkylating agent chemotherapy.

### Resistance to alkylating agent damage by ALKBH

Expression of genes in the ALKBH pathway in tissues varies^[50]^, as does the level in tumors^[51-54]^. The increased expression of ALKBH3 in some pancreatic cancers is associated with poor outcomes and reduced survival in patients, but it is unclear if high ALKBH3 levels are also associated with resistance to treatment with alkylating agents in those patients^[52]^. In breast cancer cell lines, there are also differences in the level of ALKBH3, with certain lines (e.g., MCF-7) having relatively high levels of ALKBH3 and other cell lines (e.g., Bt-474) having relatively low levels of ALKBH3^[53]^. Despite those differences in ALKBH3 levels, the contribution to alkylating agent resistance has not been evaluated. However, the accumulation of 3-meC suggests that cells with lower 3-meC are more resistant to alkylating agent treatment. Despite the varied levels of ALKBH3 in breast tumor cell lines, the survival of cells treated with methylating agents was not evaluated^[53]^. In 265 primary breast cancer samples, 72 patients showed epigenetic silencing^[53]^. ALKBH3 is also linked to prostate cancer and an alternate name is prostate cancer-1 (PCA-1)^[55,56]^. Thus, targeting ALKBH3 is a potential way to use alkylating agent therapy for prostate cancer^[55]^.

**Figure 7:**
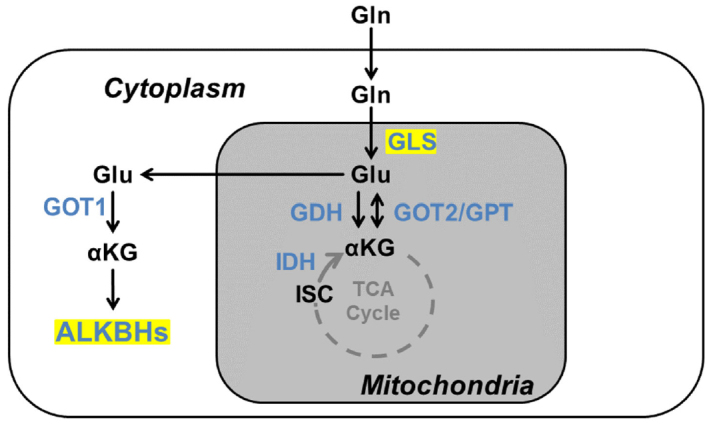
Glutamine metabolism producing 2-oxoglutarate. Enzymes are indicated in blue. ALKBHs represents ALKBH2 and ALKBH3 proteins that require αKG as a cofactor in this review. Gln: Glutamine; Glu: glutamate; αKG: α-ketoglutarate, ISC: isocitrate; TCA: tricarboxylic acid cycle; GDH: glutamate dehydrogenase; GLS: glutaminase; GOT: glutamate oxaloacetate transaminase; IDH: isocitrate dehydrogenases.

ALKBH pathway resistance can be overcome by reducing the activity of the ALKBH proteins. One way that ALKBH activities can be reduced is by the presence of mutated isocitrate dehydrogenase 1 or 2 (IDH1 or IDH2) [Figure 7]. Both those proteins are responsible for conversion of isocitrate to α-ketoglutarate. Mutated IDH1 or IDH2 is found in many cancers^[57-59]^. Mutated IDH1 or 2 leads to the production of (R)-2-hydroxyglutarate, which disrupts the activity of ALKBH^[60]^. This loss of activity renders cells more susceptible to alkylating agent treatment. Another way to restore the sensitivity of cells to alkylating agents by reducing IDH1 or IDH2 levels is to limit glutamine levels using glutaminase inhibitors^[61]^. Glutamine is a precursor of α-ketoglutarate, and inhibiting glutaminase levels can limit α-ketoglutarate levels, making cells sensitive to alkylating agents by lowering ALKBH activities. Both these methods can reduce resistance to alkylating agents in tumor cells.

### Possible resistance to alkylating agent damage by ALKBH proteins in concert with mismatch repair

Resistance is manifested when both MGMT and MMR are not functional^[39]^. O6-meG can block or impede DNA synthesis^[62]^. Both 1-meA and 3-meC can also arrest DNA synthesis, which suggests that elimination of both ALKBH pathways. Such and MMR pathways could have similar outcomes. The existence of such resistance would diminish the therapeutic outcomes for alkylating agent chemotherapy. Therefore, examining the link between the ALKBH and MMR pathways in alkylating agent damage should be a priority, because resistance to alkylating agents is often linked to MMR gene silencing.

### Interaction between the MGMT and ALKBH repair pathways

Resistance provided by MGMT and ALKBH pathways is not necessarily independent but could be cumulative or even synergistic, even though the lesions repaired by both pathways are different. Such a link could provide answers concerning resistance observed for both MGMT and ALKBH pathways separately. Investigating potential contributions of these 2 DRR mechanisms would help to understand DNA repair resistance mechanisms, which could lead to improved alkylating agent treatment efficacy.

## Conclusion

DRR is the simplest form of DNA repair, but still there are questions that are left unanswered. MGMT provides resistance to alkylation damage for O6-guanine modifications. Absence of MGMT is a positive indicator of response to alkylating agent treatment. However, the MMR pathway is needed for chemotherapy to be successful. The ALKBH pathway is less studied as compared to the MGMT pathway. Thus, we still need to address the role of MMR in ALKBH pathway repair and how the two DRR pathways work to resist alkylating agent treatments. Therefore, despite the simplicity of direct repair pathways biochemically, significant questions remain about how these pathways function to resist alkylating agent chemotherapy.

## Declarations

### Copyright

© The Author(s) 2021.
